# Classroom language during COVID-19: Associations between mask-wearing and objectively measured teacher and preschooler vocalizations

**DOI:** 10.3389/fpsyg.2022.874293

**Published:** 2022-11-09

**Authors:** Samantha G. Mitsven, Lynn K. Perry, Christian M. Jerry, Daniel S. Messinger

**Affiliations:** ^1^Department of Psychology, University of Miami, Coral Gables, FL, United States; ^2^Department of Psychology, Indiana University, Bloomington, IN, United States; ^3^Department of Electrical and Computer Engineering, University of Miami, Coral Gables, FL, United States; ^4^Department of Pediatrics, University of Miami, Coral Gables, FL, United States; ^5^Department of Music Engineering, University of Miami, Coral Gables, FL, United States

**Keywords:** language, vocalizations, preschool children, hearing loss, COVID-19, phonemic diversity, objective measurement, face-masks

## Abstract

During the COVID-19 pandemic, mask-wearing in classrooms has become commonplace. However, there are little data on the effect of face-masks on children’s language input and production in educational contexts, like preschool classrooms which over half of United States children attend. Leveraging repeated objective measurements, we longitudinally examined child and teacher speech-related vocalizations in two cohorts of 3.5–4.5-year-old children enrolled in the same oral language classroom that included children with and without hearing loss. Cohort 1 was observed before COVID-19 (no face-masks, *N* = 20) and Cohort 2 was observed during COVID-19 (with face-masks; *N* = 15). Vocalization data were collected using child-worn audio recorders over 12 observations spanning two successive school years, yielding 9.09 mean hours of audio recording per child. During COVID-19 teachers produced a higher number of words per minute than teachers observed prior to COVID-19. However, teacher vocalizations during COVID-19 contained fewer unique phonemes than teacher vocalizations prior to COVID-19. Children observed during COVID-19 did not exhibit deficits in the duration, rate, or phonemic diversity of their vocalizations compared to children observed prior to COVID-19. Children observed during COVID-19 produced vocalizations that were longer in duration than vocalizations of children observed prior to COVID-19. During COVID-19 (but not before), children who were exposed to a higher number of words per minute from teachers produced more speech-related vocalizations per minute themselves. Overall, children with hearing loss were exposed to teacher vocalizations that were longer in duration, more teacher words per minute, and more phonemically diverse teacher speech than children with typical hearing. In terms of production, children with hearing loss produced vocalizations that were longer in duration than the vocalizations of children with typical hearing. Among children observed during COVID-19, children with hearing loss exhibited a higher vocalization rate than children with typical hearing. These results suggest that children’s language production is largely unaffected by mask use in the classroom and that children can benefit from the language they are exposed to despite teacher mask-wearing.

## Introduction

Learning language poses a formidable challenge for young language learners, requiring them to integrate information across both auditory and visual domains in order to effectively process the language occurring in their surrounding environment (Rosenblum, 2008; Lewkowicz and Hansen-Tift, 2012). The onset of the COVID-19 pandemic, which resulted in the widespread use of face-masks, altered the language-learning landscape as face-masks both occlude important visual information as well as alter the quality of the auditory signal. The potential barriers to language imposed by face-mask use may be amplified by the context in which language is occurring. For example, noisy language environments such as those that occur in preschool classrooms may compound difficulties accessing language occurring through a mask (Crandell and Smaldino, 2000; Klatte et al., 2013; Grieco-Calub, 2021). Further, face-mask use may differentially affect children with hearing loss who often experience inconsistent or distorted access to acoustical information compared to their peers with typical hearing. The current study leveraged repeated objective measurements of classroom vocalizations to compare the quantity and phonemic diversity of children’s language input and production prior to the onset of the COVID-19 pandemic to the quantity and phonemic diversity of children’s language input and production while wearing face-masks during COVID-19.

### Speech perception and transmission with face-masks

The challenges that face-masks impose on vocal interactions are two-fold. First, face-masks may degrade the auditory signal, particularly for high-frequency speech sounds which can make subsequent perception of those sounds challenging (Corey et al., 2020; Goldin et al., 2020; Vos et al., 2021). Difficulties with speech perception may be compounded by an absence of facial cues from the speaker which normally serve to enhance speech perception when the auditory signal is degraded (Erber, 1969; Lalonde and Holt, 2015). Together, these challenges can both affect a speaker’s ability to effectively transmit speech to a listener as well as a listener’s ability to comprehend the speech of the speaker. In the context of interactions between caregivers and children, these difficulties may both affect the caregiver’s ability to provide responsive feedback to the child’s vocalizations as well as the child’s ability to learn from the caregiver’s language input.

Studies examining the effects of face-masks on speech perception in adults suggest that adults are relatively skilled at recovering language presented through a face-mask as evidenced by nonsignificant differences in speech perception during opaque mask relative to no-mask conditions (Mendel et al., 2008; Atcherson et al., 2017; Magee et al., 2020; Cohn et al., 2021). Adults’ wide-range vocabularies and overall top-down knowledge of language may facilitate their ability to recuperate language presented under suboptimal listening conditions. However, for children who are still learning language and are relatively inexperienced, recovery of speech through a face-mask may pose a more formidable challenge.

As children tackle the task of learning language, they may rely on visual cues such as lip or tongue movements to learn the individual phonemes and words of their language (Teinonen et al., 2008; Weatherhead and White, 2017). Face-masks obscure children’s access to these articulatory cues which may inhibit their perception and acquisition of the fine-grained details of language. In the laboratory, Singh et al. (2021) found that there was no difference in 2-year-old infants’ ability to locate a target word referent when the target word was presented by a speaker wearing an opaque mask compared to a speaker with no mask. However, the consequences of face-mask use on children’s language experiences in noisier, more naturalistic contexts remain unclear.

### Language development during preschool

The preschool years are the period of development that is marked by the most language growth. During this period, children experience a vocabulary explosion in which they go from producing approximately 500 words at 2.5 years of age to producing 10,000 words by the age of 6 (Bloom, 2002; Herschensohn, 2007). Children during this period use their vocabulary knowledge to produce utterances containing three or more words, which may include articles (a, the), auxiliary verbs (is, are, have), and pronouns (him, her) (Conti-Ramsden and Durkin, 2012). The preschool years are also marked by advances in children’s understanding of the pragmatics of language. For example, children during this time begin to master the cues that signal turn-taking in conversations (Matthews, 2014). Children are also becoming more proficient at understanding and producing longer periods of discourse (Griffin et al., 2004). The development of this constellation of oral language skills, including receptive and expressive vocabulary, grammatical knowledge, and discourse comprehension and production during the preschool period is a strong predictor of children’s later literacy skills (Storch and Whitehurst, 2002; Kendeou et al., 2009). Thereby, characterizing children’s oral language skills during preschool as well as the extent to which the language-learning environment (i.e., mask-wearing in the classroom) may alter the development of these skills is important for documenting sources of variability that may ultimately lead to more or less optimal literacy outcomes.

In addition to the preschool years being a particularly important *time* for language development, preschool itself is a particularly important *context* for early language input. High-quality interactions with teachers in preschool can support children’s development of oral language skills both over the course of a day and over the course of a year (or many years). Same-day associations between the rate of input that children are exposed to from teachers and their own rate of talking and between the phonemic diversity of children’s language input and production in the classroom have been observed. For example, children who are exposed to a higher rate of speech from teachers on a given day, produce a higher rate of vocalizations themselves on that same day (Perry et al., 2018; Mitsven et al., 2021). Similarly, children who are exposed to more phonemically diverse vocalizations from teachers, produce more phonemically diverse vocalizations themselves (Mitsven et al., 2021). In addition to same-day associations between teacher speech and children’s speech, language input from teachers also exhibits long-range associations with children’s language and literacy skills. Teachers’ increased use of strategies to both elicit and extend children’s talk during teacher-child conversations in the classroom has been shown to be associated with larger vocabulary gains for children over the course of the school year (Cabell et al., 2015). Exposure to variegated vocabulary from teachers in preschool not only predicts children’s growth in syntactic comprehension (Huttenlocher et al., 2002) and oral language skills (Gámez, 2015) over the course of the school year, but it also predicts children’s long-range reading comprehension abilities into elementary school (Dickinson and Porche, 2011). Given the impact of teacher input both on children’s language production in the moment and its consequences for children’s developing language skills over time, we were particularly interested in examining whether face-mask use in the classroom as a result of the COVID-19 pandemic altered the quantity and phonemic diversity of children’s language input from teachers. Further, we were interested in examining whether previously documented associations between children’s language input and production would be weakened as a result of mask-wearing in the classroom.

Notably, there are individual differences between children in the timing in which they achieve language-learning milestones and this is true both for typically developing children and children at risk for language delays, such as children with hearing loss. The wide degree of heterogeneity in the timing of children’s acquisition of language skills means that children enrolled in a single preschool classroom likely vary in their language level at any given time. This is particularly true for children who are enrolled in inclusive classroom settings, where children with disabilities are enrolled alongside children without disabilities (typically developing children). Differences in children’s oral language skills may elicit differential input from teachers (Dykstra et al., 2013). Further, children may be differentially affected by the language-learning environment. For example, children at risk for language delays may be particularly susceptible to negative consequences associated with language presented under suboptimal listening conditions, such as language presented through a mask. We discuss this possibility below as it pertains to children with hearing loss.

### Potential difficulties of mask use for children with hearing loss

The association between mask-wearing and children’s language input and production has been a topic of interest as it relates to all children but particularly children with hearing loss who may be disproportionately affected by mask use. Children with hearing loss experience an impaired auditory system and even with aided hearing, neither cochlear implants or hearing aids provide the same resolution as the auditory system of an individual with typical hearing. For example, while hearing aids amplify the volume of children’s residual hearing they reduce access to high-frequency speech sounds (Stelmachowicz et al., 2001, 2002). Cochlear implants allow auditory input to be transmitted directly to the auditory nerve, however, cochlear implant users typically have difficulty with frequency discrimination, particularly for sounds in the high-frequency region, which can impact speech perception and comprehension (Macherey and Carlyon, 2014). Face-masks, which reduce the intensity of high-frequency speech sounds, may exacerbate the difficulties that are already imposed by hearing devices in perceiving high-frequency speech sounds for children with hearing loss (Goldin et al., 2020). Further, as face-masks occlude the bottom half of a speaker’s face, they impede children’s ability to engage in speech reading, a strategy used by children with hearing loss to access spoken language (Kyle et al., 2013).

Indeed, adults with profound to severe hearing loss are better able to perceive speech when it is accompanied by visual cues such as those afforded by transparent face-masks or in the absence of a face-mask in comparison to speech that is presented without visual cues (i.e., with an opaque mask; Atcherson et al., 2017). Adults with hearing loss also exhibit increased deficits in speech perception when masked speech is presented in noisy conditions compared to their peers with typical hearing (Mendel et al., 2008). General delays in oral language production accompanied by noisy environments, hearing device limitations, and the absence of visual articulatory cues may contribute to substantial difficulties in recovering degraded speech for children with hearing loss. As such, children with hearing loss may be differentially affected by masked language input occurring in the classroom.

### Current study

The current investigation examined whether the mandatory use of face-masks in preschool classrooms during the COVID-19 pandemic affected the duration, rate, or phonemic diversity of children’s language input and production in the classroom. We utilized a longitudinal research design in which two cohorts of children were observed 1–2 times per month over the span of 5 months in the first cohort and 4 months in the second cohort. Both cohorts of children were enrolled in a single oral language inclusion classroom where children with hearing loss are educated alongside peers with typical hearing. The oral language inclusion classroom studied was observed over two consecutive school years. One cohort of children was observed prior to the onset of the COVID-19 pandemic and the other was observed during the pandemic when mask-wearing was obligatory for teachers and children.

We first assessed the association between mask-wearing in the classroom and the duration, rate, and phonemic diversity of children’s language input and production across cohorts of children. We were interested in the hypothesis that there would be a reduction in the duration, rate, and phonemic diversity of teacher and child speech during COVID-19 when teachers and children were required to wear masks compared to before COVID-19 when masks were not worn in the classroom. We also examined whether there was an interaction between child cohort (before COVID-19 vs. during COVID-19) and child hearing status (hearing loss vs. typical hearing) in predicting children’s language input from teachers as well as their own language production. Given the focus of the curricula in the oral language inclusion classroom that was observed (discussed below in Classroom Characteristics), which emphasized listening and spoken language development for children with hearing loss in particular, we did not expect to see differences between children with and without hearing loss in the language input that they were exposed to from teachers related to child cohort. Simply put, we hypothesized that any differences in teacher input that were observed between children with and without hearing loss would be consistent across cohorts, and would neither be amplified nor diminished as a result of mask-wearing. In contrast, we hypothesized that any differences in language production between children with hearing loss and children with typical hearing may be amplified during COVID-19. Previous work has suggested that children with hearing loss may exhibit deficits in language production, including a reduction in their rate of vocalizations as well as less diverse speech compared to children with typical hearing (Fagan et al., 2014; Mitsven et al., 2021). We hypothesized that children with hearing loss may show greater difficulty with producing language while wearing a mask, and as such, we hypothesized that there may be greater differences between children with and without hearing loss during COVID-19 as compared to before.

We then asked whether children’s language input was associated with their language production. Specifically, we examined whether the duration of vocalizations that children were exposed to from teachers was associated with the duration of their own vocalizations, whether the rate of children’s language input was associated with the rate of their own language production, and whether the phonemic diversity of children’s language input was associated with the phonemic diversity of their language production. In line with previous work which has documented associations between the language input that children receive from teachers in the classroom and their own language production, we hypothesized that children’s language input would positively predict their own language production (Perry et al., 2018, 2022; Mitsven et al., 2021). Next, we investigated whether the strength of the associations between children’s language input and their language production differed between children that were observed prior to and during the COVID-19 pandemic. We hypothesized that associations between children’s language input from teachers and their own language production might be weakened for children observed during COVID-19 because mask-wearing may affect the intelligibility of teacher speech. During periods of mask-wearing, a given amount of teacher speech may be noisier and thereby contain less signal. It is the signal (intelligible speech) that drives child speech in our hypothesized model. If there is no intelligible speech during periods of mask-wearing, we would expect that there would not be any effect of teacher language input on children’s language production. When there is less intelligible teacher language input, we would expect less of an effect on children’s language production along with increased opportunities for other factors to affect children’s language production. Put another way, reduced teacher intelligibility may lead to other factors, including peer language input, individual differences between children, or daily fluctuations, affecting children’s language production more than teacher language input.

## Materials and methods

### Participants

Two cohorts of children were observed over two successive academic years in a single preschool classroom for children with and without hearing loss. The first cohort (Cohort 1) was observed prior to the onset of the COVID-19 pandemic between October 2019 and February 2020. The second cohort (Cohort 2) was observed in their classroom during the COVID-19 pandemic when mask-wearing was obligatory for both teachers and children. Children observed during COVID-19 (Cohort 2) were observed between March and June 2021 while both children and teachers wore cloth or surgical earloop masks in the classroom.^1^ Thirty-five total children participated, including 16 children with hearing loss (9 in Cohort 1) who wear cochlear implants (CIs) or hearing aids (HAs) and 19 children with typical hearing (11 in Cohort 1; Table 1 for the full demographic information for each participant, Supplementary Table 1 for demographic information summarized by cohort). Children’s mean age at study enrollment was 42.82 months (SD = 3.55) for children before COVID-19 (Cohort 1) and 49.58 months (SD = 4.44) for children during COVID-19 (Cohort 2). The average time between hearing device activation and study onset was 27.45 months (SD = 7.06) for children with CIs and 29.73 months (SD = 12.19) for children with HAs (Table 1). Of the 35 children, 24 were Hispanic (18 White, 5 Unknown or not reported, 1 multiracial) and 11 were non-Hispanic (3 White, 5 Black or African American, 2 Asian, 1 multiracial). The sample included 12 girls (9 in Cohort 1) and 23 boys (11 in Cohort 1). Based on teacher report, 12 children were monolingual English learners, 20 children were bilingual English-Spanish learners, one child was a bilingual English-Portuguese learner, one child was a bilingual English-American Sign Language (ASL) learner, and one child was trilingual (English, Spanish, Portuguese). Ten children qualified to receive free or reduced-price lunch based on household income. Both cohorts included one primary teacher and two teaching assistants. One teaching assistant remained the same across both cohorts, however, the second teaching assistant and the lead teacher changed from Cohort 1 to Cohort 2. Thus, five total teachers participated. All five teachers were female and Hispanic White. The Institutional Review Board for Human Subject Research at the University of Miami approved this study. We obtained parental informed consent for each child’s participation and each teacher provided informed consent for their own participation. Across the two cohorts, only one child declined to participate (97% child consent rate) while all teaching staff in each cohort (100% teacher consent rate) participated in the study.

**Table 1:** Participant characteristics for children by Cohort.

Cohort	Child	Sex	Race/ethnicity	Free/reduced meal eligibility	Hearing status	Laterality of hearing loss	Type of hearing loss	Degree of hearing loss	Device	# of recordings	Age at study onset (mos.)	Age of CI implantation/HA fitting (mos.)	Hearing age at study onset (mos.)
Before COVID-19 (Cohort 1)	1	F	White/non-hispanic	Not eligible	HL	Bilateral	Sensorineural	Moderately severe	HA	5	43	10	33
	2	M	White/hispanic	Eligible	HL	Bilateral	Sensorineural	L: Mild to severe R: Severe to profound	CI + HA	5	51	22	28
	3	F	White/hispanic	Eligible	HL	Bilateral	Sensorineural	Profound	CI	3	39	18	21
	4	F	Unknown or not reported/hispanic	Not eligible	HL	Bilateral	Sensorineural	L: Mild sloping to moderately severe R: Mild sloping to moderate	HA	5	40	11	29
	5	M	Unknown or not reported/hispanic	Not eligible	HL	Bilateral	Sensorineural	L: Mild sloping to moderately severe R: Mild sloping to moderate	HA	5	39	15	24
	6	M	White/hispanic	Eligible	HL	Bilateral	Sensorineural	Severe rising to moderate	HA	3	41	8	34
	7	M	Black/non-hispanic	Eligible	HL	Bilateral	Sensorineural	Mild to moderate	HA	4	43	13	30
	8	F	White/hispanic	Not eligible	HL	Bilateral	Sensorineural	Severe to profound	CI	1	46	15	31
	9	M	Black/non-hispanic	Eligible	HL	Bilateral	Sensorineural	L: Mild sloping to moderately severe R: Severe	HA	2	42	37	5
	10	M	Asian/non-hispanic	Not eligible	TH	N/A	N/A	N/A	N/A	5	46	N/A	46
	11	M	White/hispanic	Not eligible	TH	N/A	N/A	N/A	N/A	5	38	N/A	38
	12	F	White/hispanic	Not eligible	TH	N/A	N/A	N/A	N/A	5	43	N/A	43
	13	M	White/non-hispanic	Not eligible	TH	N/A	N/A	N/A	N/A	5	41	N/A	41
	14	F	White/hispanic	Not eligible	TH	N/A	N/A	N/A	N/A	5	40	N/A	40
	15	F	White/hispanic	Not eligible	TH	N/A	N/A	N/A	N/A	5	44	N/A	44
	16	M	White/hispanic	Not eligible	TH	N/A	N/A	N/A	N/A	3	39	N/A	39
	17	F	Asian/non-hispanic	Not eligible	TH	N/A	N/A	N/A	N/A	5	47	N/A	47
	18	M	White/hispanic	Not eligible	TH	N/A	N/A	N/A	N/A	5	47	N/A	47
	19	F	White/hispanic	Not eligible	TH	N/A	N/A	N/A	N/A	5	40	N/A	40
	20	M	White/hispanic	Not eligible	TH	N/A	N/A	N/A	N/A	4	47	N/A	47
During COVID-19 (Cohort 2)	1	F	White/hispanic	Eligible	HL	Bilateral	Sensorineural	Mild to moderate	HA	4	48	8	41
	2	M	Unknown or not reported/hispanic	Eligible	HL	Bilateral	Sensorineural	Profound	CI	6	53	22	31
	3	M	White/hispanic	Eligible	HL	Bilateral	Sensorineural	Mild to moderate	HA	5	54	21	33
	4	M	Black/non-hispanic	Eligible	HL	Bilateral	Sensorineural	Moderately severe flat	HA	5	54	4	50
	5	M	Unknown or not reported/hispanic	Not eligible	HL	Bilateral	Sensorineural	Profound	CI	5	47	29	17
	6	M	Unknown or not reported/hispanic	Not eligible	HL	Bilateral	Sensorineural	Mild to moderately severe	HA	6	60	41	19
	7	M	More than one race/hispanic	Not eligible	HL	Bilateral	Sensorineural	Profound	CI	6	47	10	37
	8	M	Black/non-hispanic	Eligible	TH	N/A	N/A	N/A	N/A	7	49	N/A	49
	9	M	White/hispanic	Not eligible	TH	N/A	N/A	N/A	N/A	6	44	N/A	44
	10	M	White/hispanic	Not eligible	TH	N/A	N/A	N/A	N/A	5	46	N/A	46
	11	M	White/hispanic	Not eligible	TH	N/A	N/A	N/A	N/A	7	46	N/A	46
	12	M	More than one race/non-hispanic	Not eligible	TH	N/A	N/A	N/A	N/A	6	51	N/A	51
	13	M	White/non-hispanic	Not eligible	TH	N/A	N/A	N/A	N/A	7	47	N/A	47
	14	F	White/hispanic	Not eligible	TH	N/A	N/A	N/A	N/A	7	53	N/A	53
	15	F	Black/non-hispanic	Not eligible	TH	N/A	N/A	N/A	N/A	7	46	N/A	46

HL, hearing loss; TH, typically hearing; HA, hearing aids; CI, Cochlear implant. For children who had separate implantation/fitting dates for each of their hearing devices, the first implantation/fitting date was used.

### Classroom characteristics

The two cohorts of children that participated in this study were enrolled over two successive years in a single oral language inclusion classroom within a university-based preschool. The preschool implemented an English dominant oral language approach. The classroom is part of an Auditory Oral Education program which provides individualized early education, listening and spoken language intervention, audiological management, and technical support for children with hearing loss and their families. The Auditory Oral curriculum targets listening and spoken language development through daily activities such as circle time and free-play.

### Data collection

**Figure 1:**
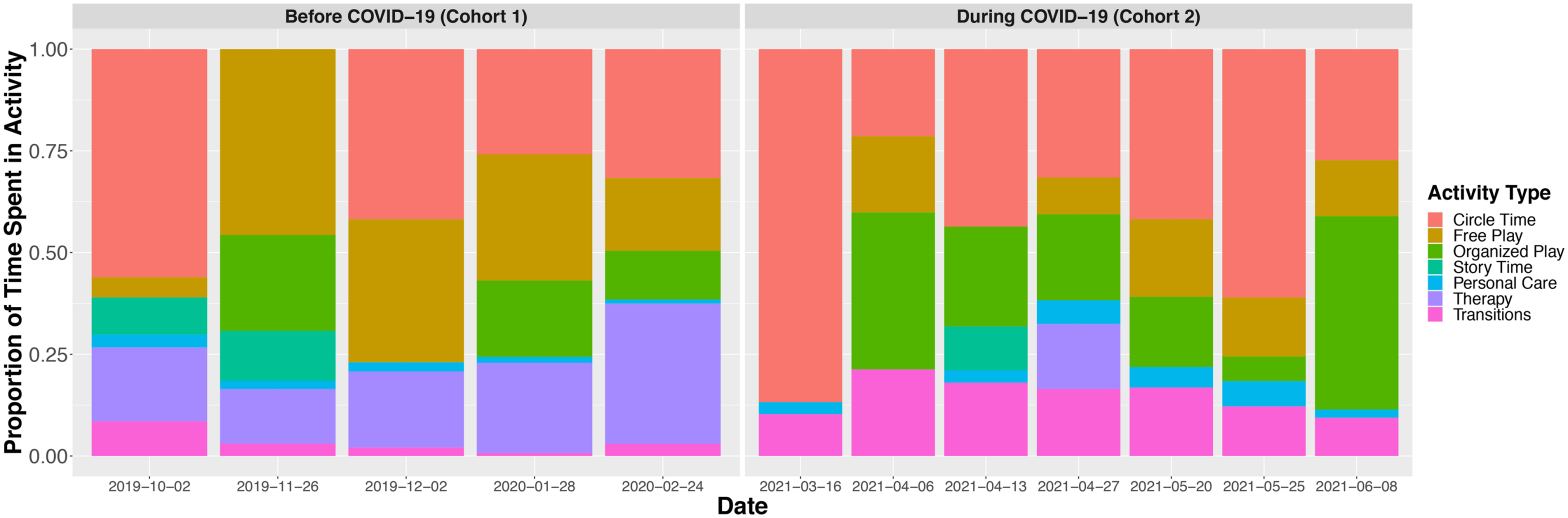
Proportion of time children spent in each activity context during each recording session. Children during COVID-19 were not required to wear masks during meal times (e.g., breakfast, snack time) or during outside play. Vocalizations that occurred during either meal time or outside play were excluded from analyses for both cohorts of children. The mean duration of meal time was 60.68 min (SD = 12.13) in the cohort observed before COVID-19 (Cohort 1) and 47.61 min (SD = 14.61) in the cohort observed during COVID-19 (Cohort 2). The mean duration of outside play was 35.16 min (SD = 18.30) in the cohort observed before COVID-19 (Cohort 1) and 56.70 min (SD = 16.45) in the cohort observed during COVID-19 (Cohort 2). Organized play includes periods in which children choose to work in stations in small groups led by a teacher doing pre-structured activities such as art projects. Personal care includes hand-washing and going to the bathroom. Not all children in the classroom attended therapy during a given recording session, thereby, the time spent in therapy reflects an average of the children who attended therapy during that recording session.

Vocalization data were collected monthly in the cohort observed before COVID-19 (Cohort 1; 5 recording sessions) and twice monthly in the cohort observed during COVID-19 (Cohort 2; 7 recording sessions) using child-worn Language ENvironment Analysis (LENA) audio recorders.^2^ Children wore a vest with a front pocket that held the LENA recorder. All consented children and teachers in attendance on recording days were recorded. On recording days, all participants were recorded for the same duration of time unless they arrived to the classroom late or left the classroom early (e.g., picked up early due to sickness). During recording sessions in Cohort 1, between 15 and 19 children and between 2 and 3 teachers were in attendance. Between 10 and 15 children and between 2 and 3 teachers were in attendance for recording sessions in Cohort 2. Children were recorded across both structured (i.e., circle time) and unstructured (i.e., free-play) activities (see Figure 1 for proportion of time children spent in each type of activity). Children observed during COVID-19 (Cohort 2) were not required to wear face-masks during meals (i.e., breakfast and snack time) or during outside play. Because our primary question of interest focused on the association between mask-wearing and children’s language input from teachers as well as their own language production, we excluded vocalizations that occurred during activities where children were not wearing masks (i.e., meal time, outside play) from analyses for both cohorts. The mean duration of recording sessions (excluding meal time and outside play) was 1.83 h (SD = 0.31; Supplementary Table 1 for descriptive statistics of recording sessions for each cohort). Children contributed a mean of 4.97 recordings (SD = 1.38), yielding 317.98 total hours of audio data to analyses (*M* = 9.09 h per child, SD = 2.49).

### Objective measurement of classroom speech-related vocalizations

#### Data processing

Audio files were analyzed using LENA SP software. LENA SP software employs maximum likelihood algorithms using pre-trained Gaussian Mixture Models (GMMs) to detect speech, distinguish between speakers, and characterize speech (Xu et al., 2008, 2009). LENA software distinguishes the speech-related vocalizations of the child wearing the LENA recorder from vocalizations made by adults and other children who are in proximity to the child wearing the recorder and provides a total count of each vocalization type (i.e., key child, adult, other child; Gilkerson et al., 2008; Xu et al., 2009). LENA software identifies speech-related vocalizations, from children and adults, as any phonemic production (e.g., from babbles to full word production). For children, phonemic production can include pre-linguistic sounds, such as cooing (resonant vowels), babbling (consonant–vowel combinations), or protophones (squeals, growls, raspberries). The minimum duration of LENA-classified child speech-related vocalizations is 600 milliseconds (ms; see Supporting Information Appendix of Oller et al., 2010). The mean duration of child speech-related vocalizations in our sample was 925.89 ms (SD = 67.34). A child speech-related vocalization is terminated if interrupted by the vocalizations of another speaker or by silence or noise of longer than 800 ms (see Supporting Information Appendix of Oller et al., 2010). LENA identifies adult speech when a vocalization of greater than 1,000 ms is spoken by either a male or female adult within an approximate 6 foot radius of the child (Irvin et al., 2013; see Supporting Information Appendix of Oller et al., 2010). LENA estimates of adult speech do not distinguish between adult speech directed toward the child wearing the recorder and adult speech directed toward other individuals that occurs in close proximity to the child wearing the recorder. We refer to LENA-classified adult vocalizations as “teacher vocalizations.” The mean duration of teacher vocalizations was 1453.48 ms (SD = 133.20). LENA distinguishes both children and adults’ speech-related vocalizations from a separate category of overlapping speech—which was not included in analyses—in which the voice of one interlocutor is accompanied by another voice or another sound source. Both children’s own speech-related vocalizations and teachers’ vocalizations were derived from the child-worn recorders.

#### Duration and rate of vocalizations

Speech-related vocalizations and their respective timestamps are reported in the LENA Interpreted Time Segments (ITS) file (Xu et al., 2008). Using the ITS file, we calculated vocalization duration by subtracting the start time from the end time of each vocalization. The durations of all vocalizations for each vocalization type (child and teacher) were averaged for each recording session such that each child had a mean duration of their own speech-related vocalizations and a mean duration of the teacher vocalizations that they were exposed to for each recording session.

The ITS file was also used to sum each child’s own speech-related vocalizations and the teacher vocalizations recorded on that child’s LENA recorder. For each recording session, the rate of child speech-related vocalizations per minute was calculated as the total number of child speech-related vocalizations divided by the length of the recording in minutes. The rate of teacher vocalizations per minute was calculated using LENA’s estimate of adult word count (AWC). AWC measures the number of words spoken in each LENA-identified adult speech segment. As we did for children’s speech-related vocalizations, we divided LENA’s estimate of AWC by the recording length in minutes to calculate the rate of teacher words that each child was exposed to during each recording session. We refer to the rate of teacher input measure as “teacher word count rate.”

#### Phonemic diversity of vocalizations

LENA-identified child and teacher speech-related vocalizations were further processed using Sphinx software to identify the individual consonants and vowels present within each vocalization. While LENA software differentiated and quantified child and adult vocalizations, Sphinx provided an estimate of the phonemic composition of each of the LENA-identified vocalizations (Lamere et al., 2003). A Python script was developed to read LENA ITS files and generate audio clips from the raw audio files based on the onsets and offsets of each speech-related vocalization. The Python script submitted the individual audio clips as input to Sphinx. Sphinx estimated the number of 39 possible phonemic units from the North American English language (24 consonants and 15 vowels) that were present in each vocalization. Using Sphinx’s estimates of the consonants and vowels present within each speech-related vocalization, we calculated the phonemic diversity or number of unique consonants and vowels present within each vocalization. The vocalizations “cracker” and “baby” are illustrative. While each vocalization represents one LENA-identified speech-related vocalization, they differ in their phonemic diversity. For example, the vocalization “cracker” (/kræ.kər/) has five total phonemes, including “k,” “r,” “æ,” “k,” and “ər.” However, the “k” phoneme in “cracker” is repeated, thereby the phonemic diversity calculation, the number of *unique* phonemes, for this vocalization would be four (phonemic diversity = 4). Similarly, the vocalization “baby” (/beɪ.bi/) includes four total phonemes, “b,” “eɪ,” “b,” and “i.” Note that the “b” phoneme in “baby” is repeated, thereby, the phonemic diversity calculation for this vocalization is three (phonemic diversity = 3). When reporting phonemic diversity results, we use the terms “more” or “less” phonemically diverse to characterize whether vocalizations contain a relatively higher (or lower) number of unique phonemes.

### Reliability

Previous studies have documented high reliability between human coders’ and LENA’s classification of child and adult vocalizations occurring in preschool classrooms (Fasano et al., 2021; Mitsven et al., 2021). However, the reliability of LENA’s classification of classroom vocalizations has not been assessed while children and teachers are wearing face-masks. To assess whether LENA’s classification of child and adult vocalizations was affected by mask-wearing in the classroom, we conducted stringent reliability coding. Four trained coders blind to LENA designations re-coded 6,958 speech-related vocalizations. This reliability sample constituted approximately 2.6% of the total sample of 269,844 recorded speech-related vocalizations. Speech-related vocalizations were sampled across recording sessions for 20 children (Cohort 1: 6 children with hearing loss, 5 children with typical hearing; Cohort 2: 5 children with hearing loss, 5 children with typical hearing). The reliability sample consisted of 50% adult and 50% child speech-related vocalizations. The trained coders listened to each speech-related vocalization and classified the speaker as either a child or adult. Percent agreement and Cohen’s Kappa (*K*) were averaged across children. Comparisons between LENA and human coders on whether a vocalization was a child or adult speech-related vocalization indicated 84% mean agreement (SD = 8.79) across cohorts (*K* = 0.68, SD = 0.17). There was 83% mean agreement (SD = 11.94) between human coders and LENA in classifying speech-related vocalizations from the cohort observed before COVID-19 (Cohort 1; *K* = 0.66, SD = 0.24) and 86% mean agreement (SD = 2.99) between human coders and LENA in classifying speech-related vocalizations from the cohort observed during COVID-19 (Cohort 2; *K* = 0.71, SD = 0.06).

### Analytic approach

Analyses were conducted in R version 3.6.2 and RStudio version 1.2.5033 (R Core Team, 2019). We conducted separate linear mixed-effects models to examine associations between mask-wearing in the classroom and children’s language input from teachers as well as children’s own language production. Mixed-effects models were run through the *lmer* function in the “lme4” package of R (Bates et al., 2014). In these models, recording sessions (level 1) were nested within children (level 2). Each model included a random intercept of subject (child). Continuous vocalization variables (e.g., mean duration, rate, phonemic diversity) were mean centered within subjects to assiduously distinguish level 1 and level 2 variance (Enders and Tofighi, 2007; Hamaker and Muthén, 2020). Significance of fixed effects for all mixed-effects models was determined using the *lmertest* function in the “lme4” package of R, which employs Satterthwaite’s degrees of freedom for assessing model fit.

Beginning with features of teachers’ speech as dependent variables, we first examined whether there was an association between mask-wearing in the classroom and children’s language input from teachers. Specifically, we were interested in testing the hypothesis that features of teachers’ speech to children (mean teacher vocalization duration, teacher word count rate, teacher phonemic diversity) would be decreased during COVID-19 (Cohort 2) when teachers were wearing masks in the classroom. Three separate linear mixed-effects models were conducted with mean teacher vocalization duration, teacher word count rate, and teacher phonemic diversity as the respective dependent variables. To capture the effect of mask-wearing on children’s language input from teachers, children’s cohort (Cohort 1 vs. Cohort 2) was included as a child-level predictor at level 2 in each model. Children observed before COVID-19 (Cohort 1) served as the reference group in the cohort contrasts. To assess whether there was an association between children’s own language production and the language input that they received from teachers, individual features of children’s speech (mean child vocalization duration, child rate, child phonemic diversity) were included as time-varying predictors at level 1. Each model only included the child vocalization feature that was equivalent to the teacher vocalization feature that was being predicted. For example, in the linear mixed-effects model where the mean duration of teachers’ vocalizations was the dependent variable, the mean duration of children’s vocalizations was included as a predictor. Child hearing status was included as a child-level predictor at level 2 in each model. Children with hearing loss served as the reference group for hearing status contrasts. Time since the start of the school year (in days) was included as a predictor at level 1 in each model to account for linear changes in language input. We included time since the start of the school year (rather than chronological age) as our measure of linear change in objectively measured language because for children with hearing loss who have not had access to spoken language for the entirety of their life, chronological age is not necessarily linearly associated with language gains to the same extent as it is for children with typical hearing.^3^ As such, we used the number of days since the start of the school year (time since start of school year) to capture changes in children’s language input and production over time. Each model also included two interaction terms, one captured the interaction between children’s language production and their cohort membership in predicting teachers’ speech and the second captured the interaction between children’s cohort membership and hearing status in predicting teachers’ speech.

Next, we examined whether there was an association between mask-wearing in the classroom and children’s language production. We tested the hypothesis that features of children’s language production (mean child vocalization duration, child rate, child phonemic diversity) would be decreased during COVID-19 (Cohort 2) when children were wearing masks in the classroom. Three separate linear mixed-effects models were conducted with mean child vocalization duration, child rate, and child phonemic diversity as the respective dependent variables. As in the teacher models, predictors in the child models included cohort, individual features of teachers’ speech (mean teacher vocalization duration, teacher word count rate, teacher phonemic diversity), child hearing status, and time since start of school (in days). Two interaction terms were included in each model, one that assessed the interaction between children’s language input from teachers and children’s cohort membership in predicting children’s language production and a second term that assessed the interaction between children’s cohort membership and hearing status in predicting children’s language production.

## Results

Bivariate correlations between variables aggregated over recording sessions are reported in Table 2. The mean duration and rate of children’s vocalizations were associated as were the mean duration and rate of teachers’ vocalizations. Children who produced speech-related vocalizations that were longer in duration produced a higher number of speech-related vocalizations per minute than children who produced speech-related vocalizations that were shorter in duration (*r* = 0.71, *p* < 0.01). Similarly, children who were exposed to teacher vocalizations that were longer in duration were exposed to a higher number of teacher words per minute (*r* = 0.77, *p* < 0.01). The phonemic diversity of speech-related vocalizations was positively associated with the mean duration and rate of speech-related vocalizations for both children and teachers. Children who produced more phonemically diverse speech-related vocalizations produced vocalizations that were longer in duration (*r* = 0.55, *p* < 0.01) as well as produced a higher rate of vocalizations per minute (*r* = 0.58, *p* < 0.01). Similarly, children who were exposed to more phonemically diverse teacher vocalizations were also exposed to teacher vocalizations that were longer in duration (*r* = 0.78, *p* < 0.01) and were exposed to a higher rate of teacher words per minute (*r* = 0.42, *p* = 0.01). The mean duration of children’s speech-related vocalizations was positively associated with both the mean duration of teacher vocalizations (*r* = 0.36, *p* = 0.04) as well as the rate of teacher words (*r* = 0.35, *p* = 0.04).

**Table 2:** Correlations between and child and teacher language measures.

			1	2	3	4	5	6	7	Total *M* (SD)	Hearing loss *M* (SD)	Typical hearing *M* (SD)	Before COVID-19 (Cohort 1) *M* (SD)	During COVID-19 (Cohort 2) *M* (SD)
Child speech	1	Mean duration (ms)	1.00	0.71**	0.55**	0.36*	0.35*	−0.09	0.72**	**925.89** (67.34)	**948.11** (73.39)	**907.18** (57.16)	**888.69** (56.26)	**975.49** (45.86)
	2	Rate		1.00	0.58**	0.11	0.13	−0.14	0.40*	**3.75** (1.01)	**3.91** (1.31)	**3.61** (0.66)	**3.42** (0.87)	**4.18** (1.04)
	3	Phonemic diversity			1.00	−0.25	−0.32	−0.31	0.50**	**4.18** (0.35)	**4.07** (0.36)	**4.27** (0.32)	**4.04** (0.24)	**4.38** (0.39)
Teacher speech	4	Mean duration (ms)				1.00	0.77**	0.78**	−0.07	**1453.48** (133.20)	**1551.24** (139.15)	**1371.16** (41.62)	**1476.04** (155.52)	**1423.40** (92.58)
	5	Word count rate					1.00	0.42*	0.07	**24.61** (8.21)	**31.39** (6.80)	**18.90** (3.69)	**24.40** (8.02)	**24.89** (8.75)
	6	Phonemic diversity						1.00	−0.51**	**9.75** (0.58)	**10.04** (0.69)	**9.50** (0.32)	**10.07** (0.57)	**9.32** (0.23)
Recording sessions	7	Time since start of school year (days)							1.00	**174.95** (58.58)	**180.28** (55.07)	**170.45** (62.52)	**126.27** (17.42)	**239.84** (5.12)

Each participant contributed one value for each measure of interest, which was an average across all recording sessions (*N* = 35). Phonemic diversity, for example, reflects the mean number of unique phonemes across all vocalizations for each vocalization type (child and teacher) over all recording sessions.

* *p* < 0.05;

** *p* < 0.01. The bold values represent the average across recording sessions. One possibility would be for the “M” in the column headers to remain bold and for the “(SD)” in the column headers to be unbolded to reflect that the entries in the table where the means are bolded and the standard deviations for each measure appear in parenetheses and are unbolded.

### Language input from teachers: Associations with child-level characteristics

#### Duration of teacher vocalizations

**Figure 2:**
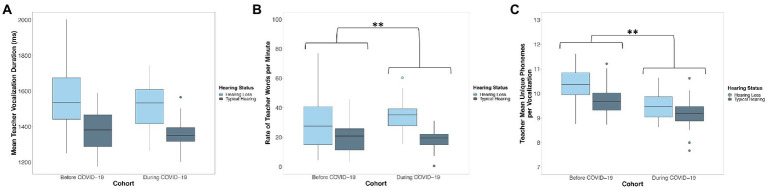
Distribution of the **(A)** mean duration, **(B)** word count rate per minute, and **(C)** phonemic diversity of children’s language input from teachers before (Cohort 1) and during COVID-19 (Cohort 2). Boxes represent the interquartile range, horizontal lines represent medians, whiskers represent error which is 1.5 times the interquartile range, and points outside of the boxes represent outliers outside of this range. Asterisks represent significant differences related to Cohort. ^*^p < 0.05, ^**^p < 0.01. Children observed during COVID-19 (Cohort 2) were exposed to more teacher words per minute but less phonemically diverse teacher speech than children observed before COVID-19 (Cohort 1). Children with hearing loss were exposed to teacher vocalizations that were longer in duration, more teacher words per minute, and more phonemically diverse teacher speech.

A linear mixed-effects model predicted the mean duration of teachers’ vocalizations from child cohort, the mean duration of children’s vocalizations, child hearing status, the number of days since the start of the school year, the interaction between cohort and the mean duration of children’s vocalizations, and the interaction between cohort and child hearing status. There was no significant difference between children observed before COVID-19 (Cohort 1; *M* = 1476.04 ms, SD = 155.52) and children observed during COVID-19 (Cohort 2; *M* = 1423.40 ms, SD = 92.58) in the mean duration of teacher vocalizations that children were exposed to (*p* = 0.555; Figure 2A; Table 3). Across cohorts, there was a significant positive association between the mean duration of children’s vocalizations and the mean duration of teachers’ vocalizations. Children who produced vocalizations that were longer in duration were exposed to teacher vocalizations that were longer in duration (*p* = 0.022, Table 3). Children with hearing loss were exposed to teacher vocalizations that were longer in duration (*M* = 1551.24 ms, SD = 139.15) than children with typical hearing (*M* = 1371.16 ms, SD = 41.62; *p* < 0.001; Figure 2A; Table 3). Neither the interaction between cohort and the mean duration of children’s vocalizations (*p* = 0.089) or the interaction between cohort and child hearing status (*p* = 0.614) were significant predictors of the mean duration of teachers’ vocalizations (Table 3).

**Table 3:** Language input from teachers: associations with child-level characteristics.

Model outcome	Model parameter	Fixed effects					
		*B*	*SE*	*t-*value	*p*	95% CI	*d*
Mean teacher vocalization duration	Cohort 1 vs. Cohort 2	−22.33	37.64	−0.59	0.555	−92.88, 50.71	−0.14
	Child vocalization duration	0.73	0.32	2.32	0.022	0.12, 1.35	0.42
	HL vs. TH	−172.54	26.60	−6.49	<0.001	−222.76, −121.87	−2.54
	Time since start of school year	−0.18	0.22	−0.81	0.420	−0.60, 0.26	−0.14
	Interaction between cohort and child vocalization duration	−0.67	0.39	−1.72	0.089	−1.43, 0.09	−0.31
	Interaction between cohort and hearing status	19.26	37.53	0.51	0.614	−52.34, 90.92	0.23
Teacher word count rate	Cohort 1 vs. Cohort 2	11.55	3.33	3.47	<0.001	5.12, 17.98	0.54
	Child rate	1.91	1.03	1.85	0.066	−0.09, 3.90	0.29
	HL vs. TH	−10.72	2.32	−4.61	<0.001	−15.21, −6.23	−0.72
	Time since start of school year	−0.07	0.02	−3.43	<0.001	−0.11, −0.03	−0.53
	Interaction between cohort and child rate	−0.31	1.25	−0.25	0.802	−2.72, 2.10	−0.04
	Interaction between cohort and hearing status	−4.33	3.26	−1.33	0.186	−10.61, 1.96	−0.21
Teacher phonemic diversity	Cohort 1 vs. Cohort 2	−1.09	0.19	−5.82	<0.001	−1.44, −0.73	−1.23
	Child phonemic diversity	0.26	0.27	0.99	0.325	−0.26, 0.78	0.17
	HL vs. TH	−0.62	0.13	−4.78	<0.001	−0.86, −0.38	−1.60
	Time since start of school year	0.002	0.001	1.73	0.085	−0.0002, 0.004	0.29
	Interaction between cohort and child phonemic diversity	0.11	0.34	0.34	0.733	−0.54, 0.77	0.06
	Interaction between cohort and hearing status	0.33	0.18	1.81	0.082	−0.02, 0.67	0.69

The table reports linear mixed effects models. Children observed before COVID-19 (Cohort 1) served as the reference in cohort contrasts. Children with hearing loss (HL) served as the reference group for hearing status contrasts. Time since start of school year was measured in days and served as a cohort-specific, time-varying predictor of teacher speech. Random intercept of subject: Teacher duration (SD = 17.38, $\sigma^{2}$=302.00), Teacher word count rate (SD = 0.00, $\sigma^{2}$=0.00), and Teacher phonemic diversity (SD = 0.08, $\sigma^{2}$=0.006). Level-1 residuals: Teacher duration (SD = 113.36, $\sigma^{2}$=12850.00), Teacher rate (SD = 10.41, $\sigma^{2}$=108.30), and Teacher phonemic diversity (SD = 0.56, $\sigma^{2}$=0.31).

#### Teacher word count rate

A linear mixed-effects model predicted the rate of teacher words per minute from child cohort, the rate of children’s vocalizations per minute, child hearing status, the number of days since the start of the school year, the interaction between cohort and the rate of children’s vocalizations, and the interaction between cohort and child hearing status. Children observed before COVID-19 (Cohort 1; *M* = 24.40, SD = 8.02) were exposed to *fewer* teacher words per minute than children observed during COVID-19 (Cohort 2; *M* = 24.89, SD = 8.75; *p* = 0.014; Figure 2B; Table 3). Across cohorts, there was not a significant association between the rate of children’s vocalizations per minute and the rate of teacher words that they were exposed to (*p* = 0.066; Table 3). Children with hearing loss were exposed to more teacher words per minute (*M* = 31.39, SD = 6.80) than children with typical hearing (*M* = 18.90, SD = 3.69; *p* < 0.001; Figure 2B; Table 3). Neither the interaction between cohort and the rate of children’s vocalizations (*p* = 0.802; Table 3) or the interaction between cohort and child hearing status were significant predictors of the rate of teacher speech (*p* = 0.186; Table 3).

#### Phonemic diversity of teacher vocalizations

The final linear mixed-effects model predicting teacher speech predicted the phonemic diversity of teachers’ vocalizations from child cohort, the phonemic diversity of children’s vocalizations, child hearing status, the number of days since the start of the school year, the interaction between cohort and the phonemic diversity of children’s vocalizations, and the interaction between cohort and child hearing status. Children that were observed before COVID-19 (Cohort 1) were exposed to teacher vocalizations that were *more* phonemically diverse (*M* = 10.07, SD = 0.57) than the teacher vocalizations that children observed during COVID-19 (Cohort 2) were exposed to (*M* = 9.32, SD = 0.23; *p* < 0.001; Figure 2C; Table 3). Across cohorts, there was not a significant association between the phonemic diversity of children’s vocalizations and the phonemic diversity of their language input from teachers (*p* = 0.325; Table 3). Children with hearing loss were exposed to teacher vocalizations that were more phonemically diverse (*M* = 10.04, SD = 0.69) than the teacher vocalizations that children with typical hearing were exposed to (*M* = 9.50, SD = 0.32; *p* < 0.001; Figure 2C; Table 3). There was not a significant interaction between cohort and the phonemic diversity of children’s language production (*p* = 0.733) or between cohort and child hearing status (*p* = 0.082) in predicting the phonemic diversity of children’s language input from teachers (Table 3).

### Children’s classroom language production

#### Duration of child vocalizations

**Figure 3:**
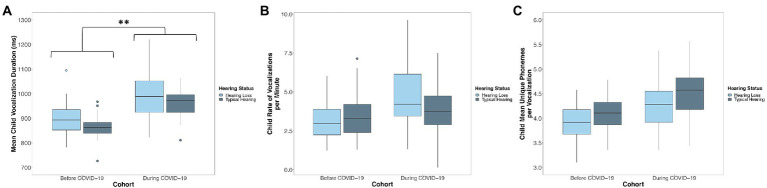
Distribution of the **(A)** mean duration, **(B)** rate of vocalizations per minute, and **(C)** phonemic diversity of children’s language production before (Cohort 1) and during COVID-19 (Cohort 2). Boxes represent the interquartile range, horizontal lines represent medians, whiskers represent error which is 1.5 times the interquartile range, and points outside of the boxes represent outliers outside of this range. Asterisks represent significant differences related to Cohort. ^*^p < 0.05, ^**^p < 0.01. Children observed during COVID-19 (Cohort 2) produced speech-related vocalizations that were longer in duration than children observed before COVID-19 (Cohort 1). Children with hearing loss produced speech-related vocalizations that were less phonemically diverse than their peers with typical hearing, but did not differ from their peers with typical hearing in the duration or rate of their vocalizations.

A linear mixed-effects model predicted the mean duration of children’s vocalizations from child cohort, the duration of teachers’ vocalizations, child hearing status, the number of days since the start of the school year, the interaction between cohort and the duration of teachers’ vocalizations, and the interaction between cohort and child hearing status. Children that were observed before COVID-19 (Cohort 1) produced vocalizations that were shorter in duration (*M* = 888.69 ms, SD = 56.26) than the vocalizations of children that were observed during COVID-19 (Cohort 2; *M* = 975.49 ms, SD = 45.86; *p* < 0.001; Figure 3A; Table 4). Across cohorts, there was not a significant association between the duration of teacher vocalizations that children were exposed to and the duration of the vocalizations that children themselves produced (*p* = 0.057; Table 4). Children with hearing loss (*M* = 948.11 ms, SD = 73.39) produced vocalizations that were longer in duration than children with typical hearing (*M* = 907.18 ms, SD = 57.16; *p* = 0.030; Figure 3A; Table 4). Neither the interaction between cohort and the mean duration of teachers’ vocalizations (*p* = 0.322) or the interaction between cohort and child hearing status (*p* = 0.488) were significant predictors of the mean duration of children’s vocalizations (Table 4).

**Table 4:** Children’s Classroom Language Production: Associations with Child-Level Characteristics.

Model outcome	Model parameter	Fixed effects					
		*B*	SE	*t-*value	*p*	95% CI	*d*
Mean child vocalization duration	Cohort 1 vs. Cohort 2	85.38	23.19	3.68	<0.001	41.64, 129.45	1.05
	Teacher vocalization duration	0.10	0.05	1.92	0.057	−0.002, 0.21	0.34
	HL vs. TH	−41.87	18.41	−2.28	0.030	−76.73, −6.99	−0.83
	Time since start of school year	−0.02	0.10	−0.24	0.814	−0.23, 0.18	−0.04
	Interaction between cohort and teacher vocalization duration	−0.09	0.09	−1.00	0.322	−0.26, 0.08	−0.17
	Interaction between cohort and hearing status	18.95	26.92	0.70	0.488	−31.95, 69.99	0.27
Child rate of vocalizations per minute	Cohort 1 vs. Cohort 2	0.83	0.54	1.52	0.132	−0.20, 1.86	0.36
	Teacher word count rate	0.02	0.01	1.44	0.153	−0.007, 0.05	0.25
	HL vs. TH	0.30	0.40	0.74	0.466	−0.47, 1.06	0.25
	Time since start of school year	0.006	0.003	1.92	0.057	−0.00001, 0.01	0.32
	Interaction between cohort and teacher word count rate	0.07	0.03	2.52	0.013	0.02, 0.12	0.44
	Interaction between cohort and hearing status	−1.21	0.58	−2.09	0.046	−2.31, −0.11	−0.77
Child phonemic diversity	Cohort 1 vs. Cohort 2	0.06	0.06	0.84	0.260	−0.13, 0.51	0.34
	Teacher phonemic diversity	0.19	0.17	1.14	0.404	−0.07, 0.17	0.14
	HL vs. TH	0.19	0.14	1.39	0.174	−0.07, 0.46	0.47
	Time since start of school year	0.001	0.0006	1.79	0.076	−0.00009, 0.002	0.30
	Interaction between cohort and teacher phonemic diversity	0.12	0.09	1.26	0.210	−0.06, 0.30	0.22
	Interaction between cohort and hearing status	0.07	0.21	0.31	0.756	−0.33, 0.46	0.11

The table reports linear mixed effects models. Children observed before COVID-19 (Cohort 1) served as the reference in cohort contrasts. Children with hearing loss (HL) served as the reference group for hearing status contrasts. Time since start of school year was measured in days and served as a cohort-specific, time-varying predictor of teacher speech. Random intercept of subject: Child duration (SD = 30.09, $\sigma^{2}$=905.60), Child rate of vocalizations per minute (SD = 0.51, $\sigma^{2}$=0.26), and Child phonemic diversity (SD = 0.27, $\sigma^{2}$=0.07). Level-1 residuals: Child duration (SD = 53.97, $\sigma^{2}$=2913.00), Child rate of vocalizations per minute (SD = 1.46, $\sigma^{2}$=2.14), and Child phonemic diversity (SD = 0.30, $\sigma^{2}$=0.09).

#### Rate of child vocalizations

**Figure 4:**
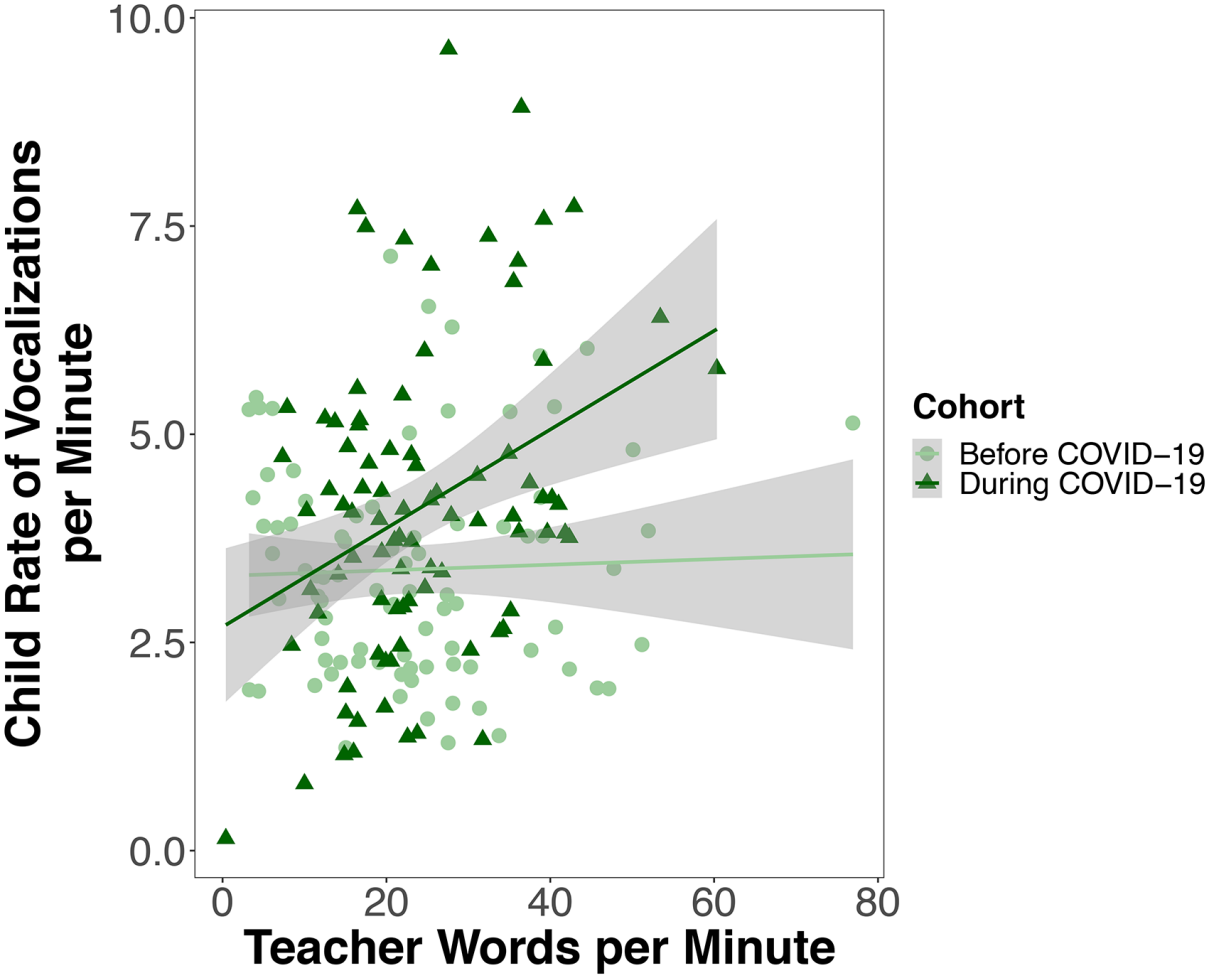
Each point represents one recording session for one child. Rate variables are expressed as number of child vocalizations or teacher words per minute. For children observed during COVID-19 (but not before), the higher the rate of teacher words per minute children were exposed to, the higher the rate of vocalizations children produced themselves.

A linear mixed-effects model predicted the rate of children’s vocalizations per minute from child cohort, the rate of teacher words per minute, child hearing status, the number of days since the start of the school year, the interaction between cohort and the rate of teacher words per minute, and the interaction between cohort and child hearing status. There was no significant difference between children observed before COVID-19 (Cohort 1; *M* = 3.42, SD = 0.87) and children observed during COVID-19 (Cohort 2; *M* = 4.18, SD = 1.04) in the rate of their vocalizations per minute (*p* = 0.132; Figure 3B; Table 4). There was not a significant association between the rate of teacher words that children were exposed to and the rate of their own speech-related vocalizations (*p* = 0.153; Table 4). There was no significant difference between children with hearing loss (*M* = 3.91, SD = 1.31) and children with typical hearing (*M* = 3.61, SD = 0.66) in the rate of vocalizations that they produced (*p* = 0.466; Figure 3B; Table 4). There was a significant interaction between child cohort and the rate of teacher words that children were exposed to in predicting the rate of children’s own speech-related vocalizations (*p* = 0.013, Figure 4; Table 4). A simple slopes analysis revealed that during COVID-19 (Cohort 2), children who were exposed to a higher rate of teacher words per minute produced a higher rate of speech-related vocalizations themselves (*B* = 0.09, *SE* = 0.02, *t* = 3.70, *p* < 0.001). However, this association was not significant for children that were observed before COVID-19 (Cohort 1; *B* = 0.02, *SE* = 0.01, *t* = 1.44, *p* = 0.153). There was also a significant interaction between child cohort and hearing status in predicting the rate of children’s speech-related vocalizations (*p* = 0.046; Table 4). A simple slopes analysis revealed that there was only a significant difference between the rate of speech-related vocalizations produced by children with hearing loss compared to children with typical hearing in the cohort of children observed during COVID-19 (Cohort 2), with children with hearing loss in this cohort producing a higher rate of speech-related vocalizations per minute than children with typical hearing (*B* = -0.91, *SE* = 0.42, *t* = −2.19, *p* = 0.038). However, there was no significant difference in the rate of speech-related vocalizations produced by children with hearing loss compared to children with typical hearing in the cohort of children observed before COVID-19 (Cohort 1; *B* = 0.30, *SE* = 0.40, *t* = 0.74, *p* = 0.466).

#### Phonemic diversity of child vocalizations

The final linear mixed-effects model predicting children’s language production predicted the phonemic diversity of children’s vocalizations from child cohort, the phonemic diversity of teachers’ vocalizations, child hearing status, the number of days since the start of the school year, the interaction between cohort and the phonemic diversity of teachers’ vocalizations, and the interaction between cohort and child hearing status. There was no difference between children observed before COVID-19 (Cohort 1; *M* = 4.04, SD = 0.24) and children observed during COVID-19 (Cohort 2; *M* = 4.38, SD = 0.39) in the phonemic diversity of their vocalizations (*p* = 0.260; Figure 3C; Table 4). There was not a significant association between the phonemic diversity of teacher vocalizations that children were exposed to and the phonemic diversity of their own vocalizations (*p* = 0.404; Table 4). There was no significant difference in the phonemic diversity of speech-related vocalizations produced by children with hearing loss (*M* = 4.07, SD = 0.36) compared to those produced by children with typical hearing (*M* = 4.27, SD = 0.32; *p* = 0.174; Figure 3C; Table 4). Neither the interaction between cohort and the phonemic diversity of teachers’ vocalizations (*p* = 0.210) or the interaction between cohort and child hearing status were significant predictors of the phonemic diversity of children’s vocalizations (*p* = 0.756; Table 4).

## Discussion

The COVID-19 pandemic and resulting safety precautions that were implemented to reduce transmission of the virus changed the learning landscape of many children. Children who were previously never exposed to language presented by caregivers wearing face coverings were constrained both to navigate language input presented through a mask and to also produce language while wearing a mask. Whether or not schools should remain open became a topic of policy debate. Although factors including decreased classroom density and increased teacher adherence to vaccination guidelines were shown to decrease simulated transmission rates within classrooms (Zhang et al., 2022), such preventative measures were not always feasible. As such, navigating language presented through a mask became a ubiquitous experience for children attending preschool throughout the pandemic as universal and consistent use of masks was one prioritized prevention strategy recommended by the Center for Disease Control and Prevention for schools providing in person instruction (CDC, 2022). However, mask mandates faced criticism as some individuals were concerned that mask use in the classroom might delay children’s developing language and social skills (Spitzer, 2020).

The current investigation aimed to characterize and compare objectively measured speech-related vocalizations collected from teachers and children prior to the onset of COVID-19 (no masks) with vocalizations collected during COVID-19 when children and teachers wore masks while in the classroom. Teachers observed during COVID-19 (Cohort 2) produced a higher rate of words per minute but also produced less phonemically diverse vocalizations than teachers observed before COVID-19 (Cohort 1). Importantly, for children that were observed during COVID-19 (Cohort 2), there was an association between the rate of teacher words that children were exposed to and the rate of their own speech-related vocalizations, an association that was not exhibited for children observed before COVID-19 (Cohort 1). Children observed during COVID-19 (Cohort 2) produced speech-related vocalizations that were longer in duration than children observed before COVID-19 (Cohort 1), however, there were no cohort differences in the rate or phonemic diversity of children’s speech-related vocalizations. Taken together, these findings suggest that mask-wearing in the classroom did not inhibit children’s language production. Further, these findings suggest that language from teachers, particularly an increased rate of words, is associated with higher rates of children’s own vocalizations, despite the challenges associated with wearing a mask.

### Associations between mask use and children’s language input

In the current study we found that children who were observed during COVID-19 (Cohort 2) were exposed to a higher rate of teacher words than children before COVID-19 (Cohort 1) were exposed to. Differences in the rate of teacher words produced with and without masks could have been a result of naturally occurring differences in the composition of the teaching staff or the composition of the classroom between cohorts. Both the lead teacher and an assistant teacher changed between the cohort observed before COVID-19 (Cohort 1) and the cohort observed during COVID-19 (Cohort 2), and some evidence suggests individual differences in the quality of teacher-child interactions are related to teacher characteristics (e.g., teacher education, years of experience, teacher burn out) (Hestenes et al., 2008; Irvin et al., 2013). The composition of the classroom also changed with the cohort observed before COVID-19 (Cohort 1) containing a higher number of children (*N* = 20) compared to the cohort observed during COVID-19 (Cohort 2; *N* = 15), which resulted in a lower teacher-child ratio prior to COVID-19. A lower teacher to child ratio prior to COVID-19 may have resulted in children having fewer opportunities to be exposed to language from teachers, a pattern that has been observed in inclusive classroom settings with low teacher-child ratios (Irvin et al., 2013).

Beyond differences between cohorts in the teaching staff and class size, it is also possible that we observed a higher rate of teacher words during COVID-19 (Cohort 2) because teachers in this cohort may have repeated themselves in order to be heard while wearing a mask. Of note, repetition in language input can be helpful for children’s language learning (Schwab and Lew-Williams, 2016; Wang et al., 2020). Repetitions are likely especially helpful for children with hearing loss who may require additional repetitions to encode words and build lexical representations given the degraded nature of speech transmitted by hearing devices. Future work utilizing transcriptions of teacher speech with and without masks is needed to disentangle whether higher rates of teacher speech while wearing masks is due to teachers’ use of repetitions, whether the repetitions are associated with mask-wearing, and whether the repetitions facilitate language learning (perhaps especially for children with hearing loss).

Despite producing more frequent language, teachers observed during COVID-19 (Cohort 2) produced vocalizations that were less phonemically diverse than the vocalizations of teachers that were observed before COVID-19 (Cohort 1). While teachers during the pandemic produced vocalizations that were less phonemically diverse, they did not differ from teachers observed prior to the pandemic in the mean duration of their vocalizations. It is possible that teachers during the pandemic were using “clear speech,” a speaking style that enhances speech intelligibility in suboptimal listening conditions, such as when speakers are wearing masks (Yi et al., 2021). The use of clear speech has been shown to benefit listeners, including children with and without hearing loss (Smiljanic and Sladen, 2013). Speakers produce fewer syllables when using clear speech compared to when they are speaking conversationally (Smiljanić and Bradlow, 2005). A decrease in syllable production would result in a decrease in the production of the sounds that make up syllables, phonemes. Thereby, if teachers during the pandemic were utilizing clear speech to enhance the intelligibility of their utterances, this could have resulted in an overall decrease in the number and potentially the diversity of phonemes that they were producing. Producing utterances slowly is another core feature of clear speech (Bradlow et al., 2003; Smiljanić and Bradlow, 2005). Teachers during the pandemic producing less phonemically diverse vocalizations of the same mean length as the vocalizations of teachers observed prior to the pandemic may have been a result of teachers during the pandemic using clear speech to increase the intelligibility of their speech while wearing a mask.

It is also possible that masks, which alter the intensity of specific types of speech sounds (e.g., high-frequency speech sounds), hampered the ability of the automated phoneme detection algorithms that we utilized through Sphinx processing software, to detect specific phonemes. Decreased sensitivity to detect specific phonemes could have resulted in the decreased phonemic diversity exhibited by teachers during the pandemic. However, we did not observe a deficit in the phonemic diversity of children’s language production during the pandemic, suggesting that there was not a systematic deficit in Sphinx’s ability to detect phonemes occurring while individuals were wearing masks. Finally, teachers observed during the pandemic may have adopted strategies beyond verbal communication, including physically getting down to the child’s level and utilizing increased gaze or pointing cues as a way of garnering children’s attention or as alternative ways of communicating meaning to bypass the potential barriers presented by mask use.

### Associations between mask use and children’s language production

Importantly, we did not observe a negative impact of mask use on children’s own language production. There were no significant differences between children who were observed prior to the pandemic and children that were observed during the pandemic in the rate or phonemic diversity of their speech-related vocalizations. In fact, children who were observed during COVID-19 (Cohort 2) produced speech-related vocalizations that were longer in duration than children who were observed before COVID-19 (Cohort 1). Notably, children who were observed during COVID-19 (Cohort 2) were observed from March to June of 2021, beginning approximately 1 year after the initial widespread implementation of mask use within the U.S. As such, children may have adopted compensatory strategies for being heard while wearing a mask. For example, children observed during the pandemic could have been committing greater vocal effort while wearing a mask, a behavior that has been reported by adults when wearing opaque masks (Ribeiro et al., 2020). Alternatively, children may have been asked to or spontaneously repeated themselves in order to be heard. If children were consistently repeating themselves, we would have expected to see an increased rate of language production in children observed during COVID-19 (Cohort 2), a pattern that we did not observe. A further possibility is that teachers were maintaining a quieter classroom environment overall to ensure that children were being heard while wearing masks.

### Associations between language input and production

Children’s language production in the classroom while interacting with both teachers and peers is directly associated with their end-of-year receptive and expressive language abilities (Perry et al., 2018; Mitsven et al., 2021). Children’s language input from teachers and from peers has been shown to be indirectly associated with children’s end-of-year language abilities as mediated by children’s own vocal productions with these social partners (Perry et al., 2018; Mitsven et al., 2021). Children’s language input then serves as a mechanism for influencing children’s own language productions, which in turn serve as a mechanism for influencing their own receptive and expressive language skills. We were thereby interested in examining the extent to which children’s language input and production were associated with one another in the current study’s sample of children overall but particularly whether any observed associations were influenced by mask-wearing in the classroom. Across cohorts, children who produced longer vocalizations were exposed to teacher vocalizations that were longer in duration. We did not observe a role of child rate or child phonemic diversity in predicting teachers’ word rate or teachers’ phonemic diversity, respectively. The reciprocal associations in which children’s language input from teachers (e.g., mean teacher vocalization duration, teacher word rate, teacher phonemic diversity) was used to predict children’s language production (e.g., mean child vocalization duration, child rate, child phonemic diversity) were also not significant in the overall sample. Together, these results suggest that overall teachers may be more sensitive to the vocal behavior of children than children are to the vocal behavior of teachers, meaning that teachers may more readily adapt their vocal behavior to match that of children. Interactive coupling in which caregivers are responsive to changes in children’s behavior has been observed in other behavioral domains such as facial affect (Chow et al., 2010).

While the rate of teacher words that children were exposed to did not predict the rate of children’s own vocalizations for the sample of children overall, we did observe a significant association between the rate of teacher words that children were exposed to and children’s own rate of vocalizations for the cohort of children observed during the pandemic. As noted earlier, factors including the increased rate of vocal input from teachers, smaller class size, and a higher teacher-child ratio experienced by children observed during COVID-19 (Cohort 2) could have resulted in children observed during COVID-19 (Cohort 2) having more opportunities to engage in back-and-forth conversations with teachers compared to children observed before COVID-19 (Cohort 1). A smaller class size during COVID-19 (Cohort 2) may have also reduced the overall noise level of the classroom, which could have allowed teachers and children to be more responsive to one another despite wearing masks.

### Classroom language experiences for children with hearing loss

Although previous investigations indicate that children with hearing loss may receive caregiver input that is less complex and less diverse than children with typical hearing, we found no evidence of impoverished input for children with hearing loss in the classroom context (Goldin-Meadow and Saltzman, 2000; Fagan et al., 2014). In fact, children with hearing loss were exposed to teacher vocalizations that were longer in duration, more frequent, and more phonemically diverse than the teacher vocalizations that children with typical hearing were exposed to. Importantly, we did not find any evidence that language input was impoverished for children with hearing loss when teachers were speaking to children with or without masks. The pattern of enhanced language input directed toward children with hearing loss in the current study may be a benefit of the type of educational program that participants were enrolled in. The Auditory Oral Education program in the current study employed curriculum designed to prepare children with hearing loss to participate in general education classrooms. As such, the focus of the classroom that was studied was on developing the listening and spoken language skills of children with hearing loss. Children with hearing loss received speech-language therapy sessions provided by licensed speech-language pathologists while at school, and thereby had additional opportunities for individualized listening and spoken language intervention compared to their peers with typical hearing. A previous investigation which observed three cohorts of children enrolled in a separate classroom within the same Auditory Oral Education program studied here found no differences in the rate or phonemic diversity of the language input that children with hearing loss were exposed to compared to children with typical hearing (Mitsven et al., 2021). Taken together, these results suggest that being enrolled in an Auditory Oral Education program provides children with hearing loss opportunities to be exposed to high-quality language.

The Auditory Oral Education program’s emphasis on facilitating the spoken language skills of children with hearing loss may have also contributed to the absence of evidence of impoverished language production for children with hearing loss when compared to their peers with typical hearing. Overall, children with hearing loss produced speech-related vocalizations that were longer in duration than the speech-related vocalizations of children with typical hearing and did not differ from children with typical hearing in the rate or phonemic diversity of their speech-related vocalizations. Children with hearing loss that were observed during COVID-19 (Cohort 2) produced speech-related vocalizations at a higher rate than children with typical hearing, while there was no difference between children with and without hearing loss before COVID-19 (Cohort 1). Together, these results suggest that children with hearing loss observed during COVID-19 were vocalizing similarly to their peers with hearing loss that were observed prior to the pandemic.

### Limitations and future directions

The current study utilized a between-cohort rather than within-cohort comparison. As such, differences between children and teachers observed before COVID-19 (Cohort 1) and children and teachers observed during COVID-19 (Cohort 2) could be attributed to individual differences in language input and production. Similarly, differences in the composition of the two cohorts (e.g., ratio of boys to girls, ratio of teachers to children, ratio of children with hearing loss to those without) could also be at play. Future work employing within-cohort comparisons of activities where subjects were required to wear masks compared to activities where they were not required to wear masks could further elucidate the impact of mask-wearing on children’s language input and production in the classroom.

While the current investigation utilized dense behavioral data collected over 318 total hours of audio recording, the small sample size (*N* = 35 children) is noteworthy. The sample sizes for each cohort, *N* = 20 for the cohort observed before COVID-19 (Cohort 1) and *N* = 15 for the cohort observed during COVID-19 (Cohort 2), reflected the total amount of children enrolled in a single oral language inclusion classroom over two successive school years. Nevertheless, future work exploring the impact of mask-wearing in the classroom would benefit from sampling of multiple classrooms and increased sample sizes which would increase the statistical power for detecting group differences. It is also important to note that the current findings may not generalize to classroom language experiences outside of Auditory Oral Language Education programs. It is possible that the masked language input and production measured within the Auditory Oral Education program studied does not reflect the masked language input and production that may be observed in other types of educational programs. The services provided by the Auditory Oral Education program in the current study included listening and spoken language intervention, audiological management, parent education, and technical support. Special auditory training strategies were incorporated into the daily curriculum of the classroom that was studied to facilitate students learning to use their hearing technology (e.g., hearing aids, cochlear implants) and development of listening and spoken language skills. Classroom instruction was provided in small group activities to facilitate listening and spoken language development. The primary emphasis on listening and spoken language skills as well as the use of primarily small group instruction within the Auditory Oral Education program may differ from more general education programs which may target a broader array of communication and social skills and which may more frequently employ large group instruction. Future work is needed to better understand the association between mask-wearing and preschooler and teacher vocalizations in other educational contexts, especially in programs including children with exceptionalities other than hearing loss.

As part of the current study’s protocol we administered the Preschool Language Scales, Fifth Edition (PLS-5) which is a standardized assessment of receptive and expressive language abilities (Zimmerman et al., 2011). The onset of COVID-19 resulted in difficulty collecting end-of-year language assessments for the children observed prior to the onset of COVID-19 as children in that cohort began to attend school in a hybrid format starting in March 2020. This resulted in us only being able to assess a subset of children which did not include any of the children with typical hearing. Additionally, the end-of-year assessments that we were able to collect in the cohort observed before COVID-19 (Cohort 1) were not standardized in terms of their method of administration with some assessments being administered virtually while others were administered in person. Consequently, we were unable to examine the extent to which children’s language input and language production in the classroom were associated with their assessed language abilities, and whether these potential associations were affected by mask-wearing. Future research including a larger sample of children who have both vocalization data in the classroom as well as an assessment of their broader receptive and expressive language abilities is needed to examine the aforementioned associations.

Finally, the current study only investigated children’s language input from teachers but did not investigate whether there were associations between mask-wearing and children’s vocal interactions with peers. We did not observe any deficits in children’s language production associated with mask-wearing which suggests that children are likely able to find ways to compensate that may generalize to their vocal interactions with peers. However, mask-wearing may impose other difficulties on children’s interactions with peers such as decreasing children’s ability to identify the facial expressions of their peers. Difficulties with identifying facial expressions may make it challenging for children to pick up on subtle social cues which could lead to more conflict with peers. Future work would benefit from examining children’s interactions with both teachers and peers to better understand whether children are able to overcome the challenges of mask-wearing in their interactions with different types of social partners.

## Conclusion

Given that mask-wearing has become a new reality that has altered the context in which children are developing, we set out to objectively capture children’s *moment-to-moment* language experiences in their classroom prior to and following the onset of ubiquitous mask use during the COVID-19 pandemic. We did not observe any differences in the rate or phonemic diversity of children’s vocalizations when they were required to wear masks compared to when they were not wearing masks. In fact, children who were wearing masks produced vocalizations that were longer in duration than children who were observed without masks, suggesting that masks were not hindering children’s language production. Together these findings suggest that children can attend preschool in person and effectively communicate with teachers while remaining safe at the same time.

## Data availability statement

The data that support the findings of this study are openly available on the Open Science Framework (https://osf.io/t98jf/).

## Ethics statement

This study, which involved human participants, was reviewed and approved by University of Miami Institutional Review Board (IRB). Written informed consent to participate in this study was provided by the participants’ legal guardian/next of kin. Adult participants (teachers) provided written informed consent for themselves.

## Author contributions

SM, LP, and DM designed the study. LP and DM obtained funding. SM and CJ processed the data. SM analyzed the data and wrote the manuscript. All authors reviewed and edited the manuscript. All authors approved the submitted version.

## Funding

The study was supported by grants from the National Science Foundation (IBSS 1620294), the Institute for Education Sciences (R324A180203), and the National Institutes of Health (R01DC018542).

## Conflict of interest

The authors declare that the research was conducted in the absence of any commercial or financial relationships that could be construed as a potential conflict of interest.

## Publisher’s note

All claims expressed in this article are solely those of the authors and do not necessarily represent those of their affiliated organizations, or those of the publisher, the editors and the reviewers. Any product that may be evaluated in this article, or claim that may be made by its manufacturer, is not guaranteed or endorsed by the publisher.

## Footnotes

^1^School administrators reported to the research team that their collaborators had conducted comparisons of speech audibility under different mask wearing conditions. These comparisons suggested that surgical or cloth masks permitted more audibility than masks with transparent windows. Other research has suggested that transparent face shields may cause additional visual distortions to the speaker’s face which can hamper children’s word comprehension (Singh et al., 2021).

^2^Data were collected once per month before COVID-19 (Cohort 1) in accordance with the study timeline and data collection schedule of a grant funding data collection in multiple preschools. During COVID-19 (Cohort 2), research activities were focused on the current classroom. Overall, the number of recording sessions before COVID-19 (Cohort 1; 5 recording sessions) compared to during COVID-19 (Cohort 2; 7 recording sessions) and the time period in which observations occurred (a 5 month period in Cohort 1 and a 4 month period in Cohort 2) were similar in the two cohorts.

^3^Children’s mean chronological age over the course of observation was significantly and positively associated with the mean number of days since the start of the school year for the range of observations, *r* = 0.58, *p* < 0.01.
