# Supplementary material for: Classroom language during COVID-19: Associations between mask-wearing and objectively measured teacher and preschooler vocalizations
Source: Front Psychol. 2022 Nov 9;13:874293. doi: 10.3389/fpsyg.2022.874293 (PMC9682284; doi:10.3389/fpsyg.2022.874293)
Supplement: Supplementary file 1 [file Data_Sheet_1.PDF]

Supplementary Material

Supplementary Table 1

Cohort	Observation period	# of recording sessions	Mean duration of recording sessions in hours (SD)	Total # of hours of audio data	TH *n*/ HL *n*	CI *n*/ HA *n*	Female *n*/ Male *n*	Monolingual *n*/ Bilingual *n*/ Trilingual *n*	Mean age in months (SD)	Mean age when child received CI/HA in months** (SD)	Mean hearing age in months *** (SD)
1	October 2019-February 2020	5	1.96 (.29)	166.85	11/9	3/7*	9/11	6/13/1	42.82 (3.55)	16.50 (8.91)	26.11 (9.07)
2	March 2021-June 2021	7	1.74 (.49)	151.13	8/7	3/4	3/12	6/9/0	49.58 (4.44)	19.36 (13.06)	32.44 (11.48)
Overall Sample	———————————	12	1.83 (.31)	317.98	19/16	6/11*	12/23	12/22/1	45.72 (5.17)	17.75 (10.61)	28.88 (10.35)

Notes. TH—Typical Hearing; HL—Hearing Loss; CI—Cochlear Implant; HA—Hearing Aids. *One child in Cohort 1 had both a CI and a HA. **For children who had two hearing devices with separate implantation/fitting dates, the first implantation/fitting date was used for determining the age at which they received their CI or HA. ***Hearing age quantifies the time that children with hearing loss have had access to spoken language via a hearing device.
